# 

**Published:** 1851-11

**Authors:** 


WHOLE SERIES—VOL. VIII, NO. IV.

Vol. IV.] NOVEMBER, 1851. [No. 4.

### THE NORTH-WESTERN MEDICAL AND SURGICAL JOURNAL.

EDITED BY JOHN EVANS, M.D.,

PROFESSOR OF OBSTETRICS &C. IN RUSH MED. COL.; MEMBER OF THE AM. MED. ASSOCIATION; ONE OF THE PHYSICIANS TO THE ILL. GEN. HOSPITAL; FORMERLY SUP'T OF THE IND. HOSPITAL FOR THE INSANE, ETC., ETC.

CHICAGO AND INDIANAPOLIS:
PRINTED BY JAS. J. LANGDON,
161 Lake street, Chicago.
1851.

ENLARGED SERIES—VOL. VI, NO. IV.

